# A 250 m Direct Time-of-Flight Ranging System Based on a Synthesis of Sub-Ranging Images and a Vertical Avalanche Photo-Diodes (VAPD) CMOS Image Sensor

**DOI:** 10.3390/s18113642

**Published:** 2018-10-27

**Authors:** Yutaka Hirose, Shinzo Koyama, Motonori Ishii, Shigeru Saitou, Masato Takemoto, Yugo Nose, Akito Inoue, Yusuke Sakata, Yuki Sugiura, Tatsuya Kabe, Manabu Usuda, Shigetaka Kasuga, Mitsuyoshi Mori, Akihiro Odagawa, Tsuyoshi Tanaka

**Affiliations:** Panasonic Corporation, 1 Kotari-yakemachi, Nagaokakyo City, Kyoto 617-8520, Japan; koyama.shinzo@jp.panasonic.com (S.K.); ishii.m@jp.panasonic.com (M.I.); saitou.shigeru001@jp.panasonic.com (S.S.); takemoto.masato3@jp.panasonic.com (M.T.); nose.yugo@jp.panasonic.com (Y.N.); inoue.akito@jp.panasonic.com (A.I.); sakata.yusuke@jp.panasonic.com (Y.S.); sugiura.yuuki@jp.panasonic.com (Y.S.); kabe.tatsuya@jp.panasonic.com (T.K.); usuda.manabu@jp.panasonic.com (M.U.); kasuga.shigetaka@jp.panasonic.com (S.K.); mori.mitsuyoshi@jp.panasonic.com (M.M.); odagawa.a@jp.panasonic.com (A.O.); tanaka.tsuyoshi@jp.panasonic.com (T.T.)

**Keywords:** avalanche photodiode, vertical avalanche photodiode, time-of-flight, CMOS image sensor, photon counting, ranging

## Abstract

We have developed a direct time-of-flight (TOF) 250 m ranging Complementary Metal Oxide Semiconductor (CMOS) image sensor (CIS) based on a 688 × 384 pixels array of vertical avalanche photodiodes (VAPD). Each pixel of the CIS comprises VAPD with a standard four transistor pixel circuit equipped with an analogue capacitor to accumulate or count avalanche pulses. High power near infrared (NIR) short (<50 ns) and repetitive (6 kHz) laser pulses are illuminated through a diffusing optics. By globally gating the VAPD, each pulse is counted in the in-pixel counter enabling extraction of sub-photon level signal. Depth map imaging with a 10 cm lateral resolution is realized from 1 m to 250 m range by synthesizing subranges images of photon counts. Advantages and limitation of an in-pixel circuit are described. The developed CIS is expected to supersede insufficient resolution of the conventional light detection and ranging (LiDAR) systems and the short range of indirect CIS TOF.

## 1. Introduction

Evolution of the autonomous vehicle system from the advanced driver assistance system (ADAS) to a fully automated one is a definitive direction of technological development [1]. In a completely man-freed system, the system is required to be intact against 3 m/s^2^ of lateral acceleration with a moving speed of 130 km/h according to the UN regulation 79. Therefore, considering the braking distance (220 m) of this speed, the system is required to range, to detect, and to recognize small objects located far ahead (>220 m). The size of the objects is as small as 10 cm corresponding to human body (arm) and to road debris such as tires and squared timbers, most commonly found on highways but very difficult to be detected. To this end, it is vital to develop a new sensing device that complements short ranging capability of forward sensing cameras and low lateral resolution of light detection and ranging (LiDAR) [2,3,4] or of radio detection and ranging (RADAR).

In this paper, we demonstrate a direct time-of-flight (TOF) backside illuminated (BSI) CMOS image sensor (CIS) based on a vertical avalanche photodiode (VAPD) pixels [5]. The pixel circuit employs a VAPD, a charge transfer gate connected to a charge accumulation capacitor, enabling one to extract sub-photon level signals [6], and a source follower output amplifier. A 44 m × 24 m wide scene at 250 m ahead is illuminated by a high power (1.2 kW peak) light pulses through diffusing optics at a repetition rate of 6 kHz, well beyond the CIS frame rate (60 fps). For ranging, each reflected pulse from an object is gated by a predetermined delay period which specifies a subrange. To cover the full range (1 m to 250 m), plural (typically 6) subranges are formed and measured. And by overlapping all subrange images, a real time depth map of full range can be generated. Thus, the present TOF-CIS is capable of ranging and imaging a 10 cm object located at as far as 250 m ahead at night. It is noted that, in contrast to conventional single photon avalanche photodiodes (SPAD) based CISs [7,8], the present VAPD based BSI CIS gives a higher aperture ratio (nearly 100%) and a smaller pixel size due to an efficient set of charge flow direction, i.e., vertical one.

The organization of this paper is as follows. In Section 2, we explain the principle of ranging by the present CIS with emphasis on operation of the in-pixel pulse counter and on a subrange synthesis method. In Section 3, experimental results of operation and ranging are presented and discussions are made. In Section 4, limitation of the in-pixel pulse counter, i.e., a maximum countable pulse number, set by charge sharing between charge storage capacitors is described in detail. We then present a preliminary results of daytime ranging under a sunlight condition of up to 60 m. Finally, we discuss a practically important problem of minimum pulse number partition into each subrange leading to a dependence of the pulse number on the subrange number, i.e., “*l*^3^-dependence”.

## 2. Principle of the VAPD Based TOF-CIS System

(1) Features of VAPD

**Figure 1:**
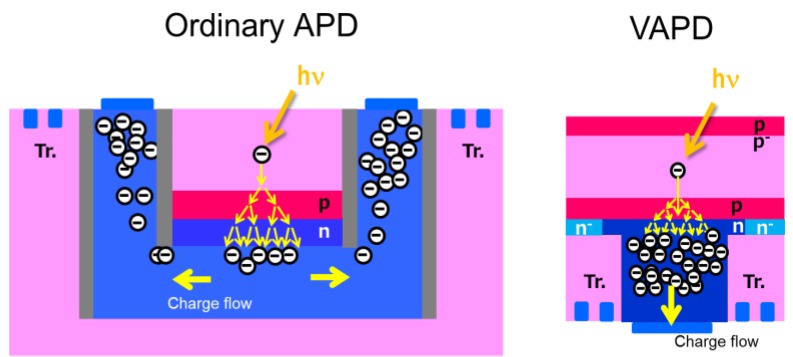
Schematics of cross sectional structures of an ordinary avalanche photodiodes (APD) pixel (**left**) and vertical avalanche photodiodes (VAPD) (**right**).

The concept of VAPD compared to an ordinary planar type APD pixel is schematically shown in Figure 1. The ordinary APD employs a ring structure in which one terminal (anode) is formed as an inner cylinder surrounded by a cathode ring. The pixel circuit is formed outside the ring structure. On the other hand, the cathode of the VAPD is formed onto the surface of the photo-conversion layer and of the PN-junction or avalanche region. The pixel circuit region can be overlapped on the PN-junction due to backside illuminated (BSI) structure. Thus, in general, VAPD has advantages of pixel size scalability and higher fill factor. The main differences are summarized in Table 1.

(2) Target of the System

**Figure 2:**
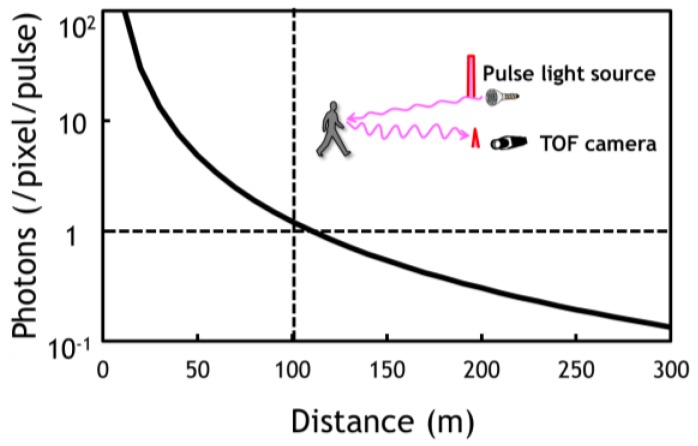
Average photon numbers per pixel per pulse reflected from an object as a function of distance (d) between a time-of-flight (TOF) system and the object showing its dependence on d^−2^. It is noted that, beyond 100 m, the average photon number falls below unity as specified by dotted lines. The assumptions made are: (1) a 10 ns pulse laser source with the peak power of 1.2 kW; (2) a field of view of 20 degrees; and (3) a 10% reflectance of an object.

The target of the full range of 250 m with the resolution of 10 cm is set under a common assumption that a vehicle would be driven on a highway with a velocity of 130 km/h necessitating over 200 m breaking distance on a maximum deceleration of 3 m/s^2^. We first estimate average photons impinging on a pixel through a TOF camera lens contained in one pulse as a function of the distance to the objects as plotted in Figure 2. A pulsed operation of a near infrared (NIR: 940 nm wavelength) laser with a 10 ns full width at half maximum (FWHM) and a 1.2 kW peak power is assumed. It is noted that the number decreases inversely proportional to square of the distance and falls far below unity over 200 m. It is also noted that the pulse width gives a depth resolution of 30 cm in this system.

(3) In-Pixel Photon Counting Circuit based on VAPD

**Figure 3:**
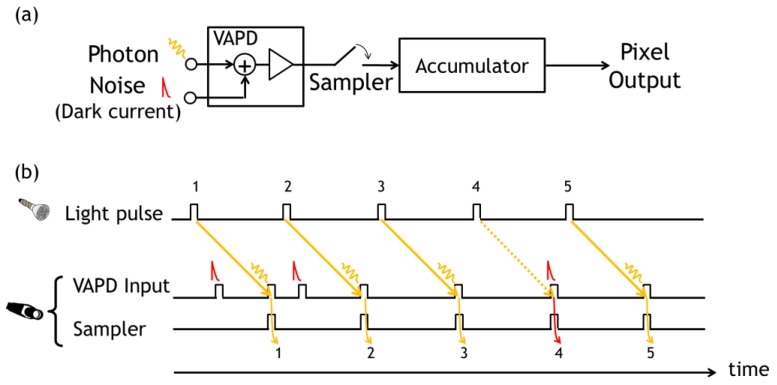
(**a**) A schematic of Photon/Noise pulse detection in VAPD equipped with a pulse sampling and accumulating circuits in the developed Complementary Metal Oxide Semiconductor (CMOS) image sensor (CIS) pixels. (**b**) A schematic timing chart of illuminating light pulse, detection and sampling. Light pulses 1, 2, 3, and 5 are successfully detected and sampled while light pulse 4 is not detected. Instead, at the timing of pulse 4, a noise pulse is sampled. By assuming that each pulse impinging on the sensor contains one or zero photon and using the notations of analysis, the primary parameters used in the analysis from Equation (1) to Equation (4) are calculated as follows. The number of sampling pulses *n* is 5. The number of detected photon pulses *k* is 4. The average number of detected photon per sampling, *λ_ph_*, is 0.8 and the average number of detecting dark pulse per sampling *λ_dc_* is 0.2.

On account of the photon absorption quantum efficiency and of avalanche probability, the photon detection probability or the equivalent photon detection efficiency (PDE) in one pulse is far below unity. Therefore, it is necessary to accumulate pulses to judge detection of a photon reflected from an object at a specified distance with high certainty. The approach corresponds to the single photon detection and average shot imaging effective for the case with a low equivalent quantum efficiency (EQE) or PDE [9]. This repeated pulse sampling scheme is illustrated in Figure 3. Each sampling is made after predetermined delay specific to each subrange or an assumed distance with a certain depth.

In the following analysis, *n* represents the number of light pulses emitted from a source and *k* represents the number of photons detected by one pixel of a sensor during a certain period. The key parameters are the photon detection probability, *p(n,k)_ph_*, and the competing dark pulse detection probability, *p(n,k)_dc_*, both of which are considered to be the Poisson distribution. Thus, during each sampling, the probabilities of detecting a photon and a dark pulse are:

(1) $$p_{ph}=1-p{\left(1,0\right)}_{ph}=1-\exp\left(-\lambda_{ph}\right)$$

(2) $$p_{dc}=1-p{\left(1,0\right)}_{dc}=1-\exp\left(-\lambda_{dc}\right)$$

where *λ_ph_* and *λ_dc_* are, respectively, average numbers of detecting photon per sampling and that of detecting dark pulse per sampling. Both *p(n,k)_ph_* and *p(n,k)_dc_* are calculated to be binomial distribution as:

(3) $$p{\left(n,k\right)}_{ph}=\frac{n!}{\left(n-k\right)!\cdot k!}\cdot p_{ph}{}^{k}\cdot {\left(1-p_{ph}\right)}^{n-k}$$

(4) $$p{\left(n,k\right)}_{dc}=\frac{n!}{\left(n-k\right)!\cdot k!}\cdot p_{dc}{}^{k}\cdot {\left(1-p_{dc}\right)}^{n-k}.$$

**Figure 4:**
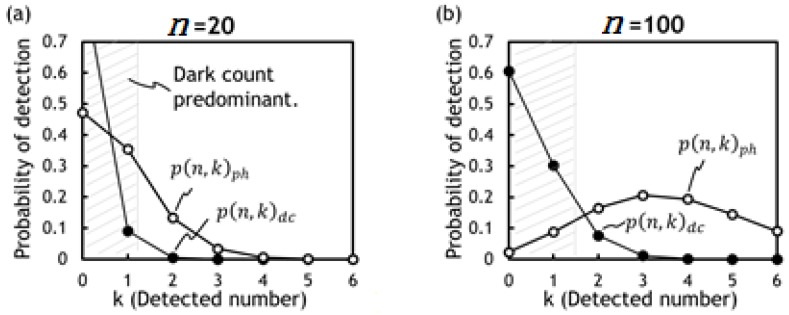
Light pulse and dark count detection probabilities for *n* = 20 (**a**) and for *n* = 100 (**b**). The hatched areas are dark count predominant regimes. As n increases from 20 to 100, both curves of *p(n,k)_ph_* and *p(n,k)_dc_* shift towards right. However, *p(n,k)_ph_* peaks at 3 meaning that 3 photon pulses are accumulated out of 100 pulses. On the other hand, dark count pulses peak at 0 with probability 0.6 and its detected probability at 3 pulses is less than 0.05. Therefore, by setting a threshold signal level of 3 pulses, the signal corresponding to photons reflected from an object is extractable.

The numerical examples of Equations (3) and (4) are calculated and plotted in Figure 4 for *n* = 20 (a) and for *n* = 100 (b). It is clearly shown that the photon signal is buried in the dark count pulses when n is 20 (a) but extracted when n is increased to 100 (b).

It is noted that similar expressions are obtained for photon detection probability of back ground light, *p(n,k)_BG_* as follows:

(5) $$p{\left(n,k\right)}_{BG}=\frac{n!}{\left(n-k\right)!\cdot k!}\cdot p_{BG}{}^{k}\cdot {\left(1-p_{BG}\right)}^{n-k}$$

where

(6) $$p_{BG}=1-p{\left(1,0\right)}_{BG}=1-\exp\left(-\lambda_{BG}\right).$$

In order to distinguish the signal pulse from background, it is necessary to count more signal pulses than those of dark current pulses.

**Figure 5:**
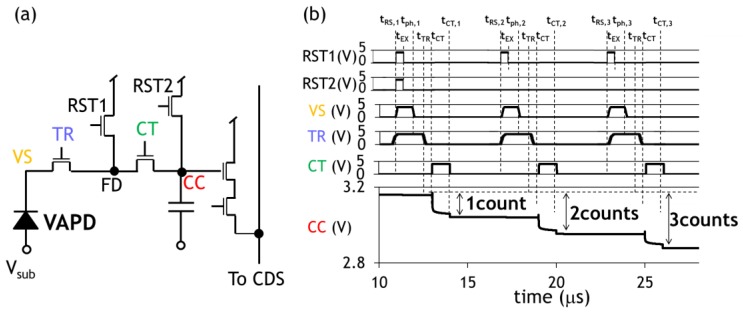
(**a**) A schematic diagram of a pixel circuit. (**b**) A simulated timing chart of the in-pixel that count and accumulate the charge output of VAPD effectively extracting photon signal as described in Figure 3b. The colored characters, VS, TR, CT, and CC, represent the nodes of VAPD cathode, transfer gate from VAPD to FD, charge transfer gate from VAPD to CC, and charge accumulation node, respectively.

In order to execute this counting operation in all the pixels synchronous to the laser pulses with 6 kHz frequency, the pixel circuit of the CIS must store signal charge with the same rate of the laser pulses. Thus, the circuit comprises the VAPD, a charge transfer (CT) gate connected to a charge accumulation capacitor (CC), and reset circuits (RST1, RST2) (Figure 5a). A result of a simulation confirming proper operation of charge accumulation is shown in Figure 5b. Initially, the cathode node VS and FD are reset to a high voltage (3.3 V) by setting the node TR and the node RST1 to HIGH (4.5 V). (t = t_RS,1_) Setting RST1 to low sets the CIS to the exposure mode. (t = t_EX_) In this simulation, at t = t_ph,1_, VS is forced to fall in order to simulate a photon detection event and subsequent avalanche multiplication in the VAPD. After detection of light and multiplication of the photo-generated electrons, the FD node is filled with large amount of charge. The exposure period ends by closing the transfer gate at time t_TR_. Then, the charge is transferred to the node CC by opening the second transfer gate CT (3.3 V) at time t_CT_. Then, a certain amount of charge corresponding to one pulse is accumulated in CC at time t_CT,1_. This counting operation is repeated until one subrange image is formed. Note the time scale of the exposure period in the simulation is order of magnitudes larger than the actual operation of the system. It is also noted that the capacitance of the CC node is much bigger (about 10 times) than that of the FD node. Such a large capacitance is formed by using an analogue metal-insulator-metal (MIM) capacitor process. Signal charge sharing between the two capacitances sets a restriction on the maximum countable pulse number as well as pulse signal nonlinearity. This sets a limitation on the ranging performance during the daytime. The effect is analyzed in Section 4. The CIS repeats this counting operation during a subrange. 

**Figure 6:**
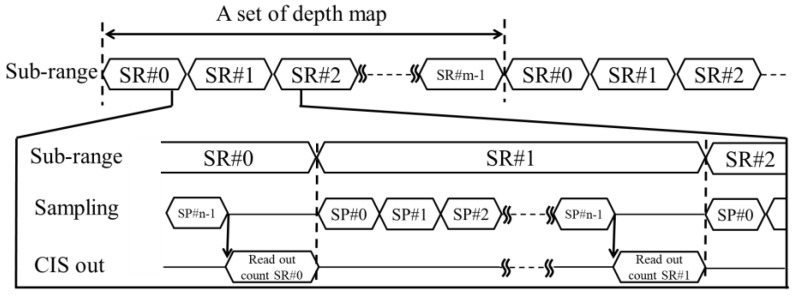
A timing diagram describing a relation between subrange sequence, light pulse sampling, and CIS output.

The number of samplings during each subrange measurement is 180. A relation between subrange sequence, light pulse sampling, and CIS output of each subrange is illustrated in Figure 6. The minimum number of sampling pulses required for distinguishing counted numbers of different subranges will be discussed in Section 4.

(4) TOF and Depth Mapping System Architecture

**Figure 7:**
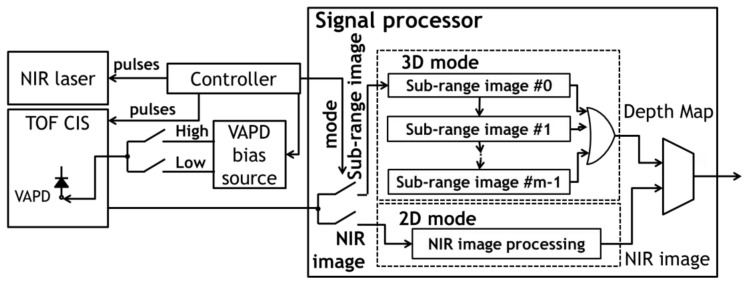
A schematic diagram of the system architecture of the TOF-CIS system.

In Figure 7, the system architecture of the developed TOF-CIS system is illustrated. The system consists of a controller, an NIR laser, a CIS, a VAPD bias source, and a signal processor. The controller globally gates the pixels synchronous to the laser pulses and switches between the 3D and the 2D modes together with the VAPD bias. In the signal processor, subrange images from TOF-CIS are overlapped to a depth map in the 3D mode while normal NIR images are output in the 2D mode.

**Figure 8:**
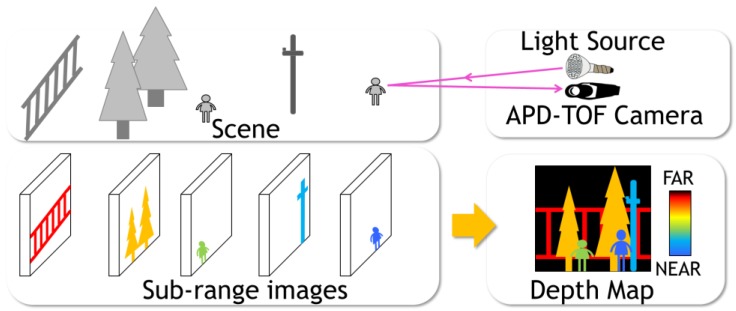
Principle of depth map construction in one frame out of plural five subrange images. Closer subrange images are colored by blue while the farther ones are colored by red.

A full range (1 m~250 m) of a scene is divided into subranges by employing multiple exposure periods in one frame. In each subrange, the charge accumulation operation is performed. Depending on the number of accumulated charge, each pixel is judged to belong or not to belong to the subrange. Thus, after a frame of exposure, all the subrange images are overlapped as illustrated in Figure 8. By giving different colors to different subranges, one is able to construct a full depth map image.

## 3. Experimental Results

(1) Device Performance

**Figure 9:**
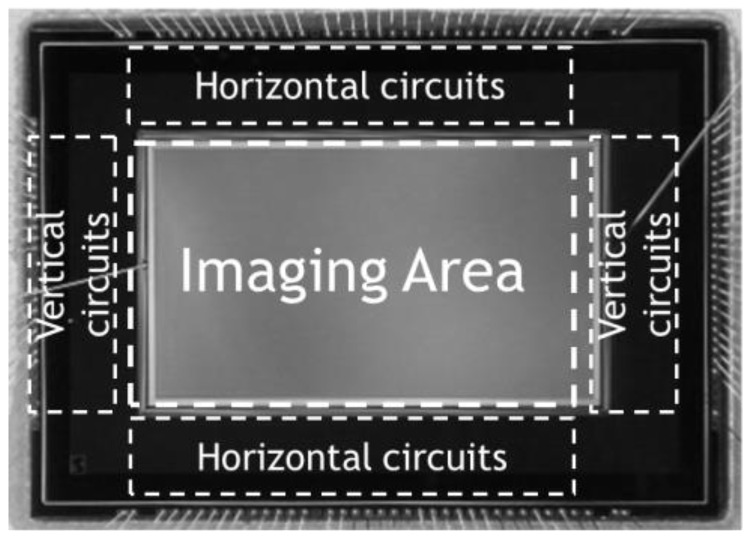
A micrograph of a developed TOF-CIS.

**Figure 10:**
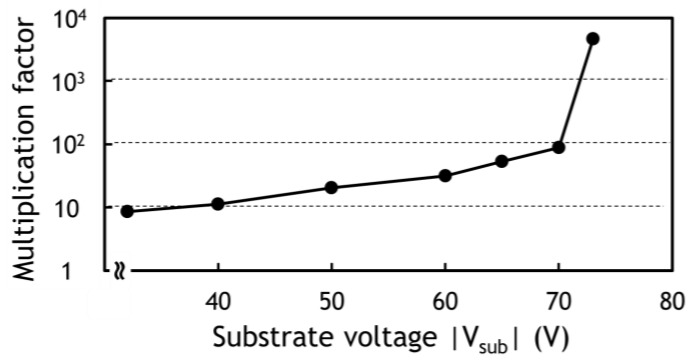
Multiplication factor of VAPD as a function of applied substrate voltage, V_sub_. It is noted that V_sub_ is actually negative since the substrate is p-type and the bias is reversed.

**Figure 11:**
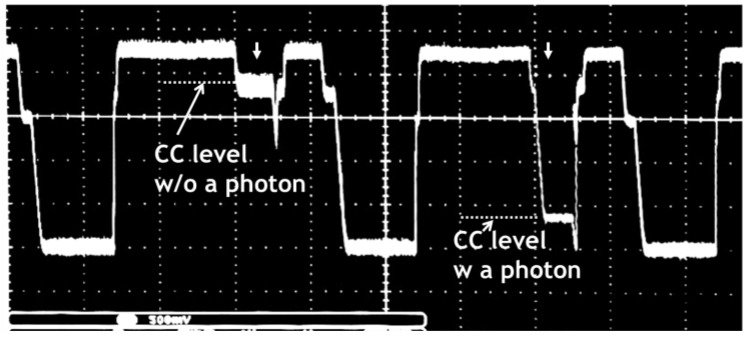
A response of VAPD outputs with and without photon detection measured at CC node. The difference of the two levels corresponding to one photon is about 1.2 V. Note the finest voltage scale is 500 mV.

A backside illuminated type (BSI) TOF-CIS with 264 K pixels (688 (H) × 384 (V)) of 11.2 μm^2^ size is fabricated with a 1-Poly 3-Metal 110 nm CMOS process as reported previously [5]. A chip micrograph of the developed CIS is shown in Figure 9 and its main specification is summarized in Table 2. The multiplication factor of the VAPD as a function of the bias voltage was measured as plotted in Figure 10. Since PDE of the present VAPD should be high at a wavelength of 940 nm, the depth of APD is much deeper than that reported in reference [5] resulting in the much higher breakdown voltage. The 3D and 2D modes were operated at >73 V with high gain (×7000) and at <70 V with low gain (×20), respectively. The amplitude of the signal corresponding to one photon was measured at the CC node of the pixel circuit as shown in Figure 11. The voltage difference between with and without photon detection is about 1.2 V which was sufficient for in-pixel light pulse counting.

(2) 250 m Range Depth Map

**Figure 12:**
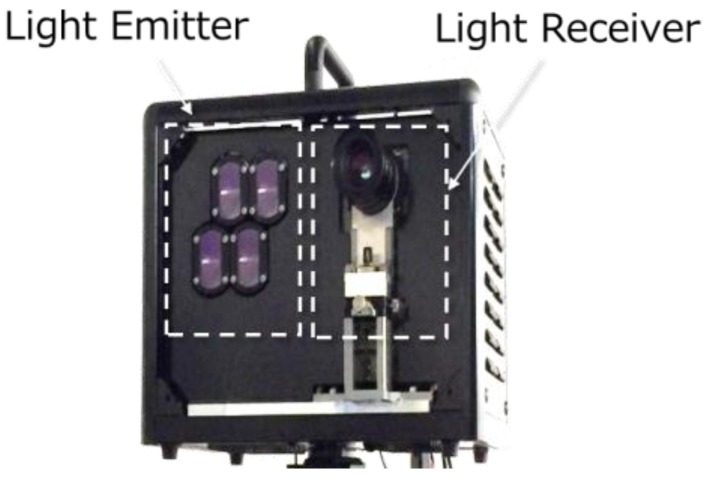
A photograph of a developed prototype TOF camera. As described in the inset, the left side is light emitter part and the right side is the receiver or CIS part.

A photograph of a developed TOF-camera system is shown in Figure 12. Four lenses on the left side are emitter (illumination) lenses each of which contains a 300 W peak pulse laser source. The right side lens is for the camera optics. Throughout the experiments presented in this work, the F number of the camera lens is set at 1.4 and the field of view is 10 degrees.

**Figure 13:**
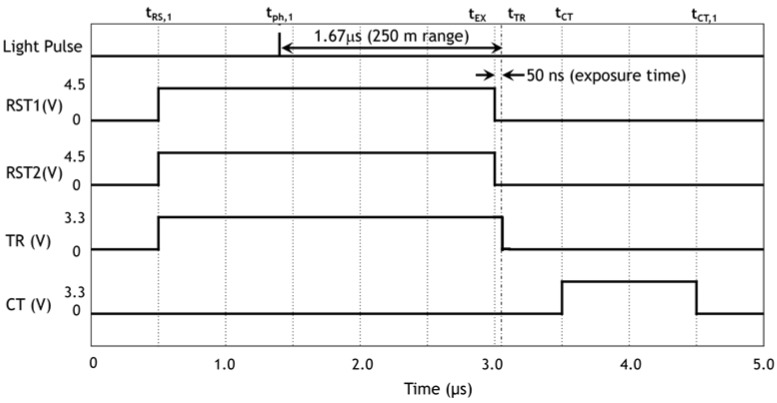
A schematic timing chart of important pulses used in the ranging experiment. The origin of time is only for convenience. The duration of laser pulse is 10 ns and the shape cannot be drawn with this scale.

We set highest priority in the fine lateral resolution (10 cm) at 250 m with full ranging (from 1 m to 250 m) capability rather than fine depth resolution with narrow ranging. The ranging experiment was performed during a night under a street light environment of 10^−1^ lux. A timing chart corresponding to 250 m ranging for most critical pulses, light pulse, RST1, RST2, TR, and CT, is plotted in Figure 13 with the timing symbols used in Figure 5b. It is noted that the exposure time, i.e., from t_EX_ to t_TR_ is 50 ns, much shorter than that shown in Figure 5 of simulation. We change the timings of t_EX_ and t_TR_ with the same amount keeping the same exposure rime as we change the subrange to be measured.

**Figure 14:**
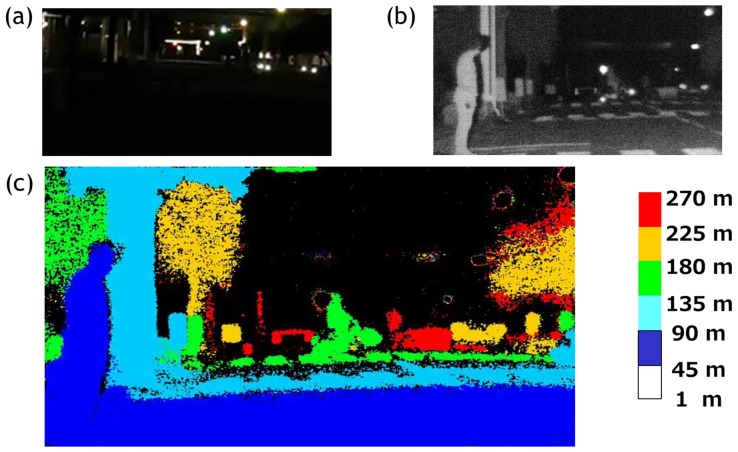
(**a**) A photograph of a street at night where the ranging experiment was performed. (**b**) An intensity image of VAPD taken at the same location. (**c**) A depth map of 270 m full range of the scene shown in (**a**,**b**). Note that a very dark scene unable to be imaged in (**a**) is clearly imaged by APD signal accumulation in (**c**) and that a 10 cm pole (red) near the center, right after a bicyclist (green colored) located at a 250 m distance is clearly resolved.

The results of the ranging experiments are shown in Figure 14. A photograph of a real dark scene taken by a digital camera is shown in the Figure 14a. For comparison, an intensity image taken by a VAPD CIS is shown in Figure 14b. Finally, a full range of 270 m depth map is obtained as shown in Figure 14c. We set six subranges, each of which is 45 m. In each subrange, we recognize a pedestrian in the first zone colored by blue, a street tree and a crosswalk colored by light blue, a cyclist on another crosswalk colored by light green, a street tree (left side) colored by yellow, and a car and a pole with 10 cm width colored by red or located at 250 m, thus demonstrating the initially specified target.

## 4. Discussions

(1) Side Effects of Charge Sharing Between FD and CC Nodes in the In-Pixel Counter

**Figure 15:**
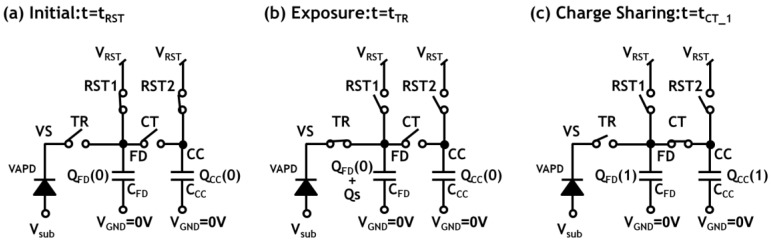
Pixel circuit diagrams during the charge transfer process. Transistors are represented by switches. The reset voltage through RST1 and RST2 are assumed to be identical, *V_RST_*.

In the following, we analyze the charge sharing effects after the charge transfer from FD to CC nodes in the in-pixel counter. Since during the charge transfer from FD to CC, TR gate is turned off, we have only to consider the capacitances of FD and CC nodes. A main sequence is illustrated in Figure 15. We use the same symbols as those of Figure 5. The parasitic capacitance of the FD node is denoted as C_FD_ whereas the capacitance of CC is *C_CC_*. At an initial time t = t_RST_ (Figure 15a), the two capacitors, C_FD_ and C_CC_, are reset by closing the switches RST1 and RST2, respectively, to the same voltage, V_RST_.

Initial charge stored in *C_FD_* and *C_CC_* are denoted as *Q_FD_*(0) and *Q_CC_*(0), respectively,

(7) $$Q_{FD}\left(0\right)=C_{FD}\cdot V_{RST}$$

and

(8) $$Q_{CC}\left(0\right)=C_{CC}\cdot V_{RST}.$$

At a time t = t_TR_ (Figure 15b), an exposure period is completed and we assume that signal charge, *Q_S_*, due to one photon detection and multiplication in VAPD is stored in *C_FD_*. Note that *Q_S_* is negative since, in the present device, signal charge is due to electrons. At this stage, therefore, the total charge in *C_FD_* is increased to:

(9) $$Q_{FD}\left(0\right)+Q_{S}.$$

When charge transfer from FD to CC is completed at t = t_CT,1_ (Figure 15c), the final charge stored in *C_FD_* and *C_CC_* are, respectively, denoted as *Q_FD_*(1) and *Q_CC_*(1). From charge conservation, the following relation holds.

(10) $$Q_{FD}\left(0\right)+Q_{S}+Q_{CC}\left(0\right)=Q_{FD}\left(1\right)+Q_{CC}\left(1\right)$$

Substituting Equations (7) and (8) into Equation (10), we obtain:

(11) $$Q_{CC}\left(1\right)=Q_{CC}\left(0\right)+rQ_{S}$$

where

(12) $$r=\frac{C_{CC}}{C_{CC}+C_{FD}}.$$

By assuming for simplicity that the amount of repeatedly generated avalanche charge is the same as the first signal and by repeating a similar procedure, we obtain the charge stored in *C_CC_* at time at t = t_CT,2_, *Q_CC_*(2) as:

(13) $$Q_{CC}\left(2\right)=Q_{CC}\left(0\right)+\left(r+r^{2}\right)\cdot Q_{S}.$$

By induction, or simply repeating this procedure, one obtains the following general expression for charge stored in *C_CC_* after *n* pulses are generated and stored.

(14) $$Q_{CC}\left(n\right)=Q_{CC}\left(0\right)+\sum_{k=1}^{n}r^{k}\cdot Q_{S}=Q_{CC}\left(0\right)+r\cdot \frac{1-r^{n}}{1-r}\cdot Q_{S}$$

**Figure 16:**
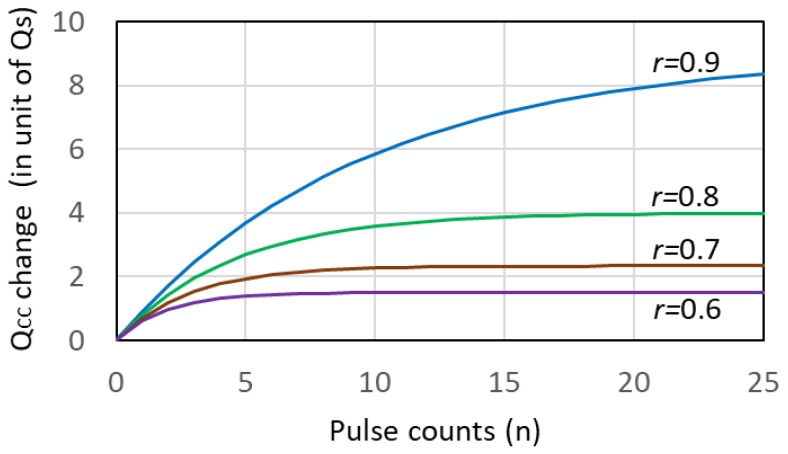
The function $r\cdot \frac{1-r^{n}}{1-r}$ appeared as the coefficient of *Q_S_* in Equation (14) showing nonlinearity with respect to *n*. For *n* larger than 10, strong deviation from linearity is observed even for *r* = 0.9.

The second term of the right hand side is the (negative) charge changed or stored in *C_CC_* after the initial value *Q_CC_*(0). We immediately notice that the stored charge is not linear with respect to the number of pulses, *n*. We plotted the function $r\cdot \frac{1-r^{n}}{1-r}$, in Figure 16 with *r* as a parameter. It should be noted that *r* should be larger in order to keep linearity. From this plot, with a typical value of *r* = 0.9, nonlinearity is significant and a proper correction (calibration) is necessary in order to count up to higher values. Because of this nonlinearity, there is a maximum of *n* practically acceptable, i.e. with a value of *r* = 0.9, the maximum is about 20.

Also, with *r* = 0.9 and *n* = 21 in Figure 16, change in *Qcc* per pulse is about 0.14 Qs, a threshold charge to be counted as valid signal. An approximate voltage value corresponding to this charge is about 10 mV across CC and 1.0 V at the FD node. It should be noted that the above analysis is valid only for Qs is finite. If Qs happened to be zero, charge is transferred to the opposite direction. Practically, it is necessary to add an element such as a diode to prevent this effect.

**Figure 17:**
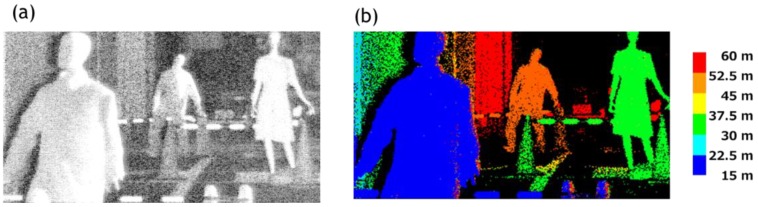
An intensity image (**a**) and a depth map (**b**) taken during daytime under a sunlight condition. The full range is 60 m and each subrange is set as 7.5 m. Pedestrians from near range to 50 m range are clearly recognizable.

The above consideration is applied to evaluation of ranging performance during daytime. In order to distinguish the ranging signal from background, the above *n_max_* must be larger than the peak counts of the background. From Equation (8), it is expressed as:

(15) $$N\cdot p_{BG}<n_{max}$$

where *N* is a total number of sampling pulses. In our preliminary experiments during the daytime, we have found the above condition is satisfied for the distance of 50 m to 60 m range in a bright sunlight environment. The result is shown in Figure 17 where pedestrians, a crosswalk, and the wall of buildings are clearly ranged. It should also be noted that against very strong background, blooming due to such light sources, and multiple paths interference effects remain to be solved.

(2) Partition of Light Pulses into the lth Subrange (*l*^3^-Dependence)

In general, the longer the distance, the more light pulses need to be counted because of the reduction in the photon numbers reflected from an object inversely proportional to the square of distance. It is practically important to partition pulse numbers into each (*l*-th) subrange, which is limited by the pulse repetition rate and by the frame rate. At first glance, such partition appears arbitrary and this would be determinable only by experimental calibration. However, by considering the dependence of the sampling probability on *l* (the number of the subrange) and a property of the binominal distribution, a simple guideline that tells the “minimum” number gives nearly *l^3^* dependence as shown below. Here, it is assumed that we only consider pulses sampled in one frame instead of the readout scheme shown in Figure 6.

First, we assume a simple situation that we had *N_l_* pulses in the *l*-th subrange and obtain the highest probability of detection at the counted number same as the subrange number, i.e., *l*. Because *l* is an integer, this corresponds to the case where the total pulse number needed is minimized. The probabilities of detecting a photon considered in Section 2-(2) is actually a function of *l* expressed as:

(16) $$p_{ph,l}=1-\exp\left(-\lambda_{ph,l}\right).$$

As discussed in Section 2-(1), the rate of arriving photons, *_ph,l_*, is proportional to inverse of the square of distance to an object or to the number of the subrange *l*. Therefore, Equation (16) is rewritten as:

(17) $$p_{ph,l}=1-\exp\left(-\frac{\lambda_{ph,1}}{l^{2}}\right).$$

The corresponding probability of detecting *k* pulses out of *N_l_* pulses in the *l*-th subrange is also modified to:

(18) $$p{\left(N_{l},k\right)}_{ph.l}=\frac{N_{l}!}{\left(N_{l}-k\right)!\cdot k!}\cdot p_{ph,l}{}^{k}\cdot {\left(1-p_{ph}\right)}^{N_{l}-k}.$$

The assumed count of *l* should occur at the peak of the probability density function (7), which in turn, is the average of this equation. By simply making use of the formula of the binominal distribution and using Equation (17):

(19) $$l=N_{l}\cdot p_{ph,l}=N_{l}\cdot \left(1-\exp\left(-\frac{\lambda_{ph,1}}{l^{2}}\right)\right).$$

For l≥3, $\frac{\lambda_{ph,1}}{l^{2}}\ll 1$ is usually satisfied. Therefore, we expand the exponential term and obtain:

(20) $$N_{l}=\frac{l^{3}}{\lambda_{ph,1}}.$$

Since it is always possible to adjust *λ_ph,1_* to be unity experimentally, we obtain the following simple expression.

(21) $$N_{l}\approx l^{3}\left(l\geq 3\right)$$

giving a cubic dependence on *l*. The numerical values calculated by Equation (21) is tabulated in Table 3. The total pulse number, 225, is close to the one obtained from experimental conditions of the pulse repetition rate of 6 kHz and the frame rate of 60 fps leading to 250 pulses/frame, which is theoretically feasible. We have confirmed, by experiments, that we only need a few pulses in the short distance. Also, for long ranging, we are using the pulse number near the number shown in Table 3. However, when *l* < 3, the above approximation does not hold and experimental conditions should be carefully analyzed. This cubic dependence significantly affects the total performance of the TOF-system, i.e., depth resolution, frame rate, and repetition rate of light source pulses.

## 5. Conclusions

We have developed a direct time-of-flight (TOF) 250 m ranging CMOS image sensor (CIS) based on a VAPD. A pixel circuit comprising a VAPD equipped with a charge transferred accumulation capacitor enables single photon signal extraction and such long distance ranging (3D). The full range was divided into plural subranges and ranging was performed by the direct TOF method in each subrange. By overlaying all the subranges images, a depth map with a 10 cm lateral resolution is realized for a full range from 1 m to 250 m. The in-pixel pulse counting circuit is described in detail. Side effects due to charge sharing between FD and a storage capacitor are explained. Especially, existence of the maximum countable pulse, *n_max_*, is shown to limit the ranging performance and in order to have a larger *n_max_*, it was shown that the storage capacitor should be larger. Finally, a simple guideline to partition the limited number of the light pulses into each subrange (*l^3^*-Dependence) was discussed. The developed CIS readily meets the requirement for ranging in a fully autonomous system described in UN Reg. 79 and is expected to supersede the insufficient resolution of the conventional light detection and ranging (LiDAR) systems and the short range of indirect CIS TOF.

## Figures and Tables

**Table 1:** Comparison of ordinary APD and VAPD.

	Ordinary APD	VAPD
Reference	[10]	This work
Type	FSI	BSI
Direction of Charge Flow	Lateral	Vertical
Pixel Size	8 m~25 m	11 m
Fill Factor	27%	>80%

**Table 2:** Specification of a developed TOF-CIS.

Process	110 nm CMOS
Metal Layers	3
Pixel	VAPD
Pixel size	11.2 μm
Pixels	688 × 384
Fill factor	>80%
Avalanche voltage	−76 V
Frame rate	60 fps
Shutter modes	Global/Rolling
Chip size	11.8 mm × 9 mm

**Table 3:** Number of light pulses necessary for each subrange.

Subrange Number (*l*)	N*_l_* = *l*^3^
1	1
2	8
3	27
4	64
5	125
Total	225

## Abbreviations

The following abbreviations are used in this manuscript:

TOF	Time-of-Flight
CMOS	Complementary Metal Oxide Semiconductor
CIS	CMOS image sensor
APD	Avalanche Photodiode
VAPD	Vertical Avalanche Photodiode
NIR	Near Infrared
SPAD	Single Photon Avalanche Photodiode
LiDAR	Light Detection and Ranging
ADAS	Advanced Driver Assistance System
FWHM	full width at half maximum
RADAR	radio detection and ranging
PDE	photon detection efficiency
EQE	equivalent quantum efficiency
BSI	Backside Illuminated
